# Supplementary material for: NcGRA7 and NcROP40 Play a Role in the Virulence of Neospora caninum in a Pregnant Mouse Model
Source: Pathogens. 2022 Aug 31;11(9):998. doi: 10.3390/pathogens11090998 (PMC9506596; doi:10.3390/pathogens11090998)
Supplement: Supplementary file 1 [file pathogens-11-00998-s001.zip › pathogens-1865666-supplementary.pdf]

Table S1. Sequences of primers used for cytokine real-time PCR (qPCR).

Gene	Forward Primer	Reverse Primer	Reference
beta-actin	5′-GGCACCACACCTTCTACAATG-3′	5′-TGGATGGCTACGTACATGGCTG-3′	45
IFN-gamma	5′-TGAAAATCCTGCAGAGCCAGAT-3′	5′-AGCTCATTGAATGCTTGGCG-3′	45
IL4	5′-TGACGGCACAGAGCTATTGATG-3′	5′-TTCTTCGTTGCTGTGAGGACG-3′	45
IL10	5′-AGGCGCTGTCATCGATTTCTC-3′	5′-TGGCCTTGTAGACACCTTGGTC-3′	45
TNFα	5′-CTGTAGCCCACGTCGTAGC-3′	5′-TTGAGATCCATGCCGTTG-3′	46

References

45. Varona, R.; Cadenas, V.; Gómez, L.; Martínez-A, C.; Márquez, G. CCR6 Regulates CD4+ T-cell–mediated acute graft-*versus*-host disease responses. *Blood* **2005**, *106*, 18–26. https://doi.org/10.1182/blood-2004-08-2996.
46. López-Pérez, I.C.; Collantes-Fernández, E.; Rojo-Montejo, S.; Navarro-Lozano, V.; Risco-Castillo, V.; Pérez-Pérez, V.; Pereira-Bueno, J.; Ortega-Mora, L.M. Effects of *Neospora caninum* infection at mid-gestation on placenta in a pregnant mouse model. *J. Parasitol.* **2010**, *96*, 1017–1020. https://doi.org/10.1645/GE-2347.1.
